# The biological significance of histone modifiers in multiple myeloma: clinical applications

**DOI:** 10.1038/s41408-018-0119-y

**Published:** 2018-08-22

**Authors:** Hiroto Ohguchi, Teru Hideshima, Kenneth C. Anderson

**Affiliations:** 1 Jerome Lipper Multiple Myeloma Center, Department of Medical Oncology, Dana-Farber Cancer Institute, Harvard Medical School, Boston, MA USA; 2 Division of Disease Epigenetics, Institute of Resource Development and Analysis, Kumamoto University, Kumamoto, Japan

## Abstract

Multiple myeloma (MM) is a clonal plasma cell disorder that is characterized by a variety of genetic alterations. Recent studies have highlighted not only the importance of these genetic events but also epigenetic aberrations including DNA methylation, histone modifications, and non-coding RNAs in the biology of MM. Post-translational modifications of histone, such as methylation and acetylation, contribute to chromatin dynamics, and are modulated by histone modifying enzymes, and dysregulation of these enzymes is implicated in the pathogenesis of cancers, including MM. Histone modifiers also have non-histone substrates and enzymatically independent roles, which are also involved in tumorigenesis. Here we review and provide comprehensive insight into the biologic significance of histone methyl- and acetyl-modifiers in MM, and further provide an overview of the clinical applications of histone modifier inhibitors, especially histone deacetylase inhibitors. These findings underline the emerging roles of histone modifiers in the pathogenesis of MM, and further highlight the possibility of novel epigenetic therapies in MM.

## Introduction

Multiple myeloma (MM) is a plasma cell malignancy characterized by clonal evolution^1^. It arises from a premalignant stage known as monoclonal gammopathy of undetermined significance (MGUS), with subsequent multistep genetic alterations resulting in progression from MGUS to smoldering MM and later to symptomatic MM^1^. Recent studies have characterized these genetic events, including chromosomal translocation/deletion and somatic mutations^2^. In addition to genetic aberrations, accumulating evidence also indicates that epigenetic changes including DNA methylation, histone modifications, and non-coding RNAs also play crucial roles in the development of this disease. For example, global DNA hypomethylation with gene-specific DNA hypermethylation has been observed in MM^3^. MM-specific microRNA (miRNA) signatures have also been reported^4^. Furthermore, dysregulation of histone modifying enzymes, such as MM SET domain (MMSET) and lysine demethylase 6A (KDM6A), have shed light on aberrant histone modifications in MM^5,6^. The functional significance of other histone modifiers is also beginning to be elucidated.

In this review, we describe the biologic roles of histone modifying enzymes in MM, especially focusing on methylation and acetylation modifiers. Where relevant, we also discuss the clinical applications of histone modifier inhibitors, especially histone deacetylase (HDAC) inhibitors. For the reviews on the other epigenetics, including DNA methylation and miRNAs in MM, we respectfully refer readers to relevant reviews^7–9^.

## Histone modifications

**Fig. 1:**
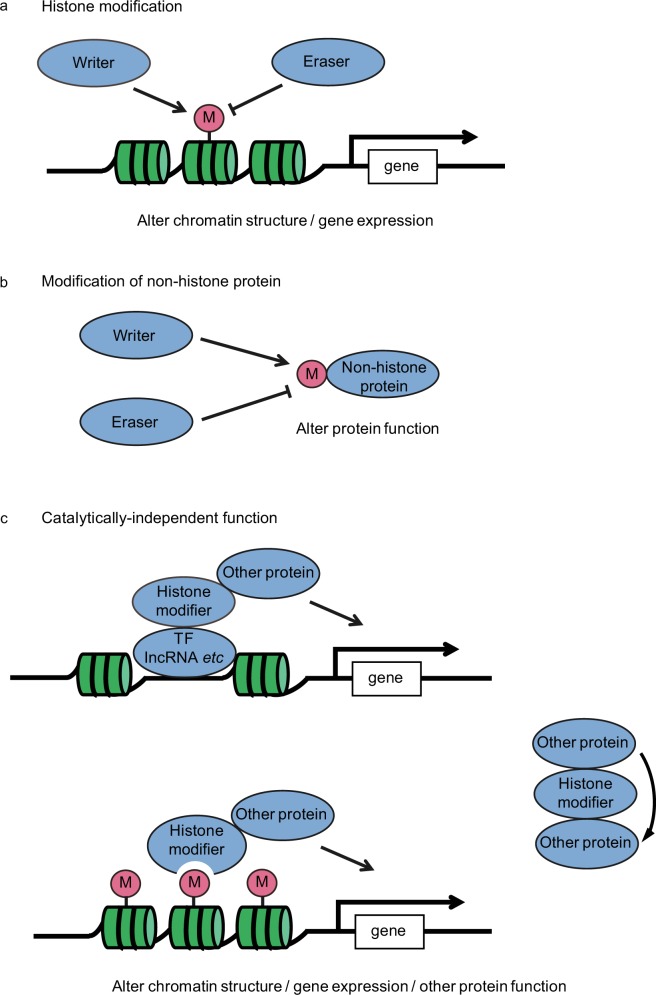
Multiple functions of histone modifiers. **a** Histone modifiers modify the basic residues of histone tail, leading to alteration of chromatin structure and gene expression. **b** Histone modifiers also modify non-histone proteins, resulting in alteration of protein function. **c** Histone modifiers can alter chromatin structure and gene expression, as well as affect other protein function in an enzymatically independent manner. TF transcription factor, lncRNA long non-coding RNA

Since Allfrey et al.^10^ demonstrated that histones can be methylated and acetylated in 1964, at least 16 classes of histone modifications including methylation, acetylation, phosphorylation, and ubiquitination have been identified^11,12^. These modifications not only alter chromatin structure, but also contribute to the recruitment of protein complexes to the modification sites, which in turn affects DNA-templated processes, such as gene expression; dysregulation of these modifications can lead to diseases, including cancers^11,13^. Hence, these modifications are strictly controlled by histone modifying enzymes, the writers and erasers of these modifications (Fig. 1). Importantly, these histone modifiers also have non-histone targets and enzymatic activity-independent functions (Fig. 1). Here we will provide an overview of histone methylation and acetylation, as well as their modifiers.

## Histone methylation

Histone methylation can occur on the side chains of arginine, lysine, and histidine residues^11,13^. Arginines are monomethylated (me1) or symmetrically or asymmetrically dimethylated (me2s/me2a), whereas lysines are mono- (me1), di- (me2) or trimethylated (me3). Histidines have been shown to be monomethylated, although this methylation has not been well defined^13^. Various basic residues throughout the histone proteins are methylated^14^: among these methylation sites, histone H3 lysine4 (H3K4), H3K9, H3K27, H3K36, H3K79, and H4K20 have been well characterized. Not only the site of the methyl–lysine residue within a histone tail but also the status of methylation is linked to gene expression status. For example, H3K9me3 is associated with gene repression, while H3K9me1 is related to gene activation^15^. In general, H3K4me3, H3K36me3, and H3K79me3 are found near the transcriptional start site of active genes, whereas H3K9me3 and H3K27me3 are at silent promoters^15^. Histone methylation status is dynamically modified by histone methyltransferases (HMTs) and lysine demethylases (KDMs) in the context of specific biological processes.

### HMTs

There are histone arginine methyltransferases and lysine methyltransferases. Arginine methyltransferases comprise nine members of the protein arginine methyltransferase (PRMT) family, which mediates three types of arginine methylation (me1/me2s/me2a)^16^. Lysine methyltransferases consist of a large family of proteins that possess the catalytic SET domain, except for one enzyme DOT1 like histone lysine methyltransferase (DOT1L)^17^. The SET domain-containing proteins are divided into six subfamilies based on sequence homology: the suppressor of variegation 39 (SUV39) family, the enhancer of zeste homolog (EZH) family, the SET domain-containing 1 (SET1) family, the SET2 family, the PR domain-containing (PRDM) family, and the SET and MYND domain-containing (SMYD) family. Lysine methyltransferases mediate mono-, di-, or trimethylation of lysine residues. Each methyltransferase has substrate specificity towards histone basic residues^17^. For example, the members of the SUV39 family methylate H3K9, while DOT1L catalyze H3K79 methylation. Importantly, HMTs also methylate various non-histone proteins, including p53 and vascular endothelial growth factor receptor 1 (VEGFR1), thereby altering their function and stability^16,18^.

### KDMs

Histone arginine demethylases have not yet been identified. KDMs are divided into two families based on catalytic mechanism: the lysine-specific demethylase (LSD) family and the Jumonji C (JMJC) family^13,19^. The LSD family catalyzes demethylation of H3K4 or H3K9 by a flavin adenine dinucleotide-dependent amine oxidation reaction^13,19^. This family can remove mono- and dimethylation from lysine residues, but not trimethylation due to the requirement of a free electron pair at the methylated lysine residue for the demethylase activity^19^. The JMJC family is characterized by the conserved JMJC domain and is comprised of more than 30 proteins; however, not all family members possess enzymatic activity. This family members are further divided into subfamilies KDM2-8, based on structural similarity^19^. Each member has substrate specificity, and KDMs against H3K4, H3K9, H3K27, H3K36, and H4K20 have been identified. The JMJC demethylases catalyze demethylation of lysine residues by a dioxygenase reaction which requires iron and α-ketoglutarate^13,19^. Due to its distinct catalytic mechanism from the LSD family, the JMJC family can demethylate trimethylated lysine residues. As is the case in HMTs, KDMs also have non-histone targets^19^, as well as catalytically independent functions^19^.

### Histone acetylation

Histones are acetylated on the side chains of lysine residues, which neutralizes lysine’s positive charge, leading to open chromatin structure by reducing interaction between histone and negatively charged DNA^11,20^. Thus, histone acetylation increases the accessibility of proteins, such as transcription factors, to promoters and enhancers, thereby mediating active gene expression. Acetylated histones also function as binding sites for numerous proteins with bromodomains, which often activates gene transcription^20^. In contrast, deacetylation of histone is associated with chromatin condensation and transcriptional repression^20^. Analogous to histone methylation, histone acetylation is reversibly controlled by two large families of enzymes: histone lysine acetyltransferases (KATs) and HDACs.

### KATs

KATs comprise two major classes of enzymes: type A and type B KATs. Type A KATs are localized in the nucleus and involved in acetylation of histones in the context of chromatin^11^. Type A KATs include the Gcn5-related *N*-acetyltransferase (GNAT) family, the MYST (MOZ, Ybf2/Sas3, Sas2, Tip60) family, and the CREB binding protein (CBP)/p300 family. Type B KATs are engaged in the acetylation of newly synthesized free histones, but not nucleosomal histones^11^. Of note, KATs serve not only as histone acetyltransferases but also as acetyltransferases of other proteins. Similar to methylation, acetylation of non-histone proteins alters their protein functions^21^. KATs also function as molecular scaffolds to recruit protein complexes^22^.

### HDACs

HDACs contain 18 enzymes, which are grouped into four classes (class I, II, III, and IV) based on structural homology^11,20^. Class I HDACs, which are homologous to yeast Rpd3, include HDAC1, 2, 3, and 8. Class II HDACs are closely related to yeast Hda1, and subdivided into two classes: class IIa (HDAC4, 5, 7, and 9) and class IIb (HDAC6 and 10). Class IV HDAC consists of only one HDAC, HDAC11, whose catalytic domain shows similarity to those of both class I and class II, but does not possess enough homology to be placed in either class. Class I, II, and IV HDACs require a zinc metal ion for their enzymatic activity^11^. Class III HDACs (Sirtuin (SIRT)1–7) share homology with yeast Sir2, and have different enzymatic mechanism from other HDACs, which does not require a zinc metal ion, but has NAD^+^ dependence^23^. Similar to KATs, HDACs have numerous non-histone substrates, including oncoproteins and tumor suppressors, and affect their functions.

## The roles of histone methylation modifiers in MM

### MMSET

MMSET (also known as NSD2/WHSC1) is one of the HMTs containing the SET domain and catalyzes addition of H3K36me2, a methyl mark associated with active chromatin^24,25^. MMSET also functions as a transcriptional repressor by interacting with sin3a, HDAC1, HDAC2, and LSD1^26^. MMSET is thought to be implicated in Wolf–Hirschhorn syndrome, a developmental disorder characterized by intellectual disability, craniofacial malformation, and heart and skeletal defects, since heterozygous deletion of MMSET is found in most patients with this syndrome^27^. MMSET is also involved in cancers. MMSET is overexpressed or somatically mutated in a variety of cancers, including MM^28–30^.

MMSET is the most extensively studied histone methyl modifier in MM because of its clinical relevance. The t(4;14)(p16;q32) is one of the most common translocations in MM, presenting in nearly 15% of patients, and is related to poor prognosis^5,31^. This translocation breakpoints on 4p16 are between genes encoding *FGFR3* and *MMSET*, resulting in juxtaposition of both genes to the immunoglobulin heavy chain enhancer^32,33^. While *MMSET* is overexpressed in all t(4;14) MM samples, *FGFR3* is expressed in only about 70% of them^5,34^. Moreover, the presence of t(4;14) is a predictor for poor prognosis regardless of *FGFR3* expression, suggesting that activation of *MMSET*, not *FGFR3*, plays a critical role in the pathogenesis of this recurrent translocation^5^.

**Fig. 2:**
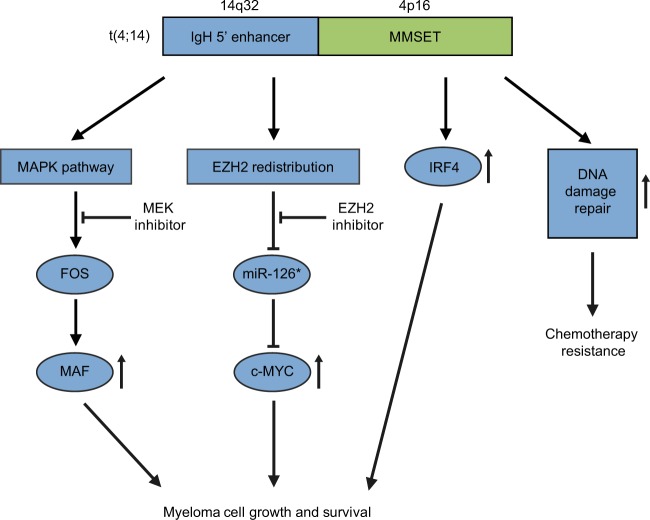
The mechanisms of MMSET-mediated MM cell growth and survival. The t(4;14)(p16;q32) results in juxtaposition of *MMSET* to the immunoglobulin heavy chain (*IgH*) 5’ enhancer, leading to aberrant *MMSET* expression. MMSET confers MM cell growth and survival via (1) *MAF* upregulation through MAPK pathway, (2) c-MYC activation through inhibition of miR-126* by EZH2, and (3) direct *IRF4* induction. MMSET also contributes to chemotherapy resistance by facilitating DNA damage repair

Recent studies have delineated the biological roles of MMSET in t(4;14) MM. Knockdown or knockout of *MMSET* induces cell cycle arrest and apoptosis, thereby reducing cell growth in t(4;14) MM cells^24,25,35,36^. Complementation of wild-type *MMSET*, but not enzymatically inactive mutant, restores cell growth in *MMSET* knockout cells^24,25^, indicating that MMSET stimulates MM cell growth depending on its methyltransferase activity. The underlying mechanisms of MMSET-mediated MM cell growth and survival are beginning to be deciphered (Fig. 2). Annunziata et al.^37^ have shown that MAF and its target genes are highly expressed not only in MM cells with *MAF* translocation, but also in those with *MMSET* translocation. They have further demonstrated that MMSET upregulates *MAF* transcription through activation of mitogen-activated protein kinase (MAPK) pathway, and that ectopic expression of *MAF* rescues t(4;14) MM cells from toxicity of *MMSET* depletion or MEK (MAPK kinase) inhibition, identifying MAF oncogene as a downstream effector of MMSET^37^. MMSET has also been shown to increase c-MYC expression by repressing expression of miR-126*, which targets 3’-untranslated region of *c-MYC* and inhibits its translation^38^. Overexpression of *MMSET* affects binding and distribution of another histone methyltransferase EZH2 across the genome, resulting in decreased global H3K27me3 level and increased H3K27me3 level at the specific loci^39^. Importantly, the miR-126* locus is one of these specific loci, and treatment with EZH2 inhibitor restores miR-126* expression, resulting in reduction of c-MYC expression and cell proliferation in *MMSET*-overexpressing cells^39^. Interferon regulatory factor 4 (IRF4) is a crucial survival factor in MM cells^40^. In t(4;14) MM cells, MMSET directly activates *IRF4* expression through binding to the *IRF4* promoter^41^. Overexpression of *MMSET* also confers chemotherapy resistance in MM cells. MMSET is involved in the DNA damage response^42,43^. Shah et al.^44^ have shown that DNA repair after treatment with DNA-damaging agents is facilitated in *MMSET*-high MM cells compared to *MMSET*-low MM cells, suggesting a reason underlying the poor prognosis of t(4;14) MM.

### EZH2

Polycomb repressive complex 2 (PRC2) maintains the silent state of target genes such as *HOX* genes through trimethylation of H3K27, contributing to development and differentiation as well as tumorigenesis^45,46^. EZH2 is a core component of PRC2 that confers the HMT activity^45,46^. Overexpression of *EZH2* is associated with progression in prostate and breast cancer^45,46^. Somatic activating mutations of *EZH2* have also been found in diffuse large B-cell lymphoma and follicular lymphoma, whereas inactivating mutations have been reported in myeloid neoplasms, suggesting context-dependent roles of EZH2 in tumorigenesis^45,46^.

EZH2 is supposed to function as an oncogene in MM. Although *EZH2* mutation has not been reported in MM, overexpression of *EZH2* is linked to disease progression and poor prognosis^47–49^. Moreover, *EZH2* is not expressed in normal bone marrow plasma cells^50^. The following studies further suggest involvement of EZH2 in MM pathogenesis. EZH2 is induced by interleukin 6 (IL-6) in IL-6-dependent MM cell lines, and expressed constitutively in IL-6-independent MM cell lines^51^. Furthermore, knockdown of *EZH2* impairs MM cell growth, whereas ectopic expression of *EZH2* induces IL-6 independence in IL-6-dependent MM cell lines^51^. c-Rel may be involved in constitutive *EZH2* expression, since *EZH2* expression is sustained by c-Rel in MM.1S, an IL-6-independent MM cell line^52^. Consistent with the notion that EZH2 is overexpressed in MM, H3K27me3 level is elevated at polycomb target genes in MM, and expression of these targets is decreased in MM compared to normal plasma cells^53,54^. This expression signature is more prominent in advanced stages of MM^53,54^. Recent studies have revealed the molecular mechanisms of EZH2-mediated MM cell growth and survival^55,56^. EZH2 interacts with the long non-coding RNA MALAT1, and EZH2 and MALAT1 cooperatively downregulate an anti-MM miRNA miR-29b by increasing H3K27me3 mark at its promoter^55^. Inhibition of EZH2 or MALAT1 induces miR-29b expression, thereby downregulating major miR-29b pro-survival targets such as SP1, CDK6, and MCL-1 and reducing MM cell growth^55^. Conversely, miR-29b inhibitor abrogates cell growth inhibition induced by EZH2 inhibitor, indicating that suppression of miR-29b mediates EZH2-driven MM cell growth and survival^55^. EZH2 and MALAT1 complex also promotes MM cell survival through epigenetic repression of *KEAP1* expression^56^. Suppression of *KEAP1* activates *NRF1* and *NRF2* expression, and then reduces endoplasmic reticulum (ER) stress-induced apoptosis by induction of proteasome gene expression^56^.

Recently, several EZH2-specific inhibitors have been developed, and pharmacologic inhibition of EZH2 by these inhibitors (E7438, UNC1999, GSK126, and EPZ005687) has been shown to exert anti-MM effects^48,54,57–60^. EZH2 inhibitors reduce global H3K27me3 level and trigger apoptosis in MM cells^48,54,57–60^. E7438 activates expression of epithelial tumor suppressor genes such as *CDH1*, *EMP1*, and *EPHB2*, although the role of these genes in MM remains unclear^57^. UNC1999 induces expression of miR-125a and miR-320c, resulting in the reduction of their targets *IRF4*, *XBP1*, and *PRDM1*^59^. UNC1999 also induces *NR4A1*, which in turn suppresses expression of *MYC*^60^. GSK126 induces the intrinsic mitochondrial apoptosis pathway, as well as decreases MM stem-like cells by blocking Wnt/β-catenin pathway^58^. GSK126 also rescues osteoblast precursors from MM-induced suppression of osteoblast differentiation, suggesting that EZH2 inhibition may also be an effective treatment of lytic bone lesions in MM^61^. EPZ005687 upregulates cell cycle control genes, resulting in cell cycle arrest^48^.

EZH2 inhibition may or may not promote drug resistance in MM. Kikuchi et al.^62^ have shown that chemotherapy agents increase global H3K27me3 level, inducing MM cell death; conversely, MM cell adhesion to bone marrow stromal cells leads to EZH2 inactivation via phosphorylation of EZH2, counteracting drug-induced H3K27 hypermethylation and MM cell death. Moreover, knockdown of *EZH2*, as well as pharmacologic inhibition of EZH2, reduce the toxicity of chemotherapy agents in MM cells, confirming that EZH2 inactivation is the underlying mechanism of cell adhesion-mediated drug resistance^62^. On the other hand, Rastgoo et al.^49^ have shown that EZH2 is elevated via downregulation of miR-138 that is a suppressor of EZH2 in drug-resistant MM cell lines, and ectopic expression of *EZH2* in parental cell lines induces drug resistance of these cell lines. Mechanistically, EZH2 directly suppresses *RBPMS* expression, which in turn activates MYC and BCL2 expression, conferring drug resistance in MM cells^49^. Conversely, restoration of *RBPMS* by forced expression of miR-138 restores the drug sensitivity in MM cells^49^.

### PRMT5

PRMT5 (also known as JBP1/SKB1) is a type II arginine methyltransferase which catalyzes mono- and symmetric dimethylation of arginine. PRMT5 regulates various cellular processes via methylation of histone and other substrate proteins, including p53 and E2F1, and deregulation of this enzyme is implicated in cancers^16^.

*PRMT5* is overexpressed in MM, and higher *PRMT5* expression is associated with poor clinical outcome^63^. Indeed, PRMT5 confers MM cell growth. PRMT5 interacts with E3 ubiquitin ligase TRIM21, and this interaction prevents TRIM21-mediated degradation of IKKβ, thereby activating nuclear factor (NF)-κB signaling^63^. Notably, PRMT5 inhibitor (EPZ015666) decreases MM cell growth by blocking NF-κB activation, suggesting a potential clinical application of this drug^63^.

### KDM3A

KDM3A (also known as JMJD1A/JHDM2A) is a member of the JMJC demethylases, which catalyze removal of H3K9me1 and H3K9me2^64^. KDM3A is implicated in many biological processes including spermatogenesis, systemic metabolism, and sex determination^65–67^. In addition, oncogenic functions of KDM3A have been reported in cancers^68,69^.

We have recently shown the biological significance of KDM3A in MM. KDM3A is highly expressed in MM, and required for MM cell survival^70^. Mechanistically, KDM3A sustains expression of *KLF2* and a MM master transcription factor *IRF4*^40^ via H3K9 demethylation at their promoters^70^. KLF2 is a transcription factor that maintains homeostasis of B cells and plasma cells^71^. We further showed that KLF2 also plays a crucial role in MM cell survival^70^. Importantly, KLF2 directly stimulates *IRF4* expression and IRF4 reciprocally activates *KLF2* expression, forming a positive autoregulatory circuit downstream of KDM3A^70^. The interaction of MM cells with bone marrow microenvironment plays an essential role in MM cell survival^72^, and KDM3A also regulates MM cell adhesion and homing to the bone marrow, further promoting MM cell growth and survival^70^. A recent report has shown KDM3A functions in hypoxic conditions^73^. *KDM3A* is induced via hypoxia-inducible factor-1α under hypoxia in MM cells, and hypoxia-induced KDM3A stimulates expression of glycolytic genes through upregulation of *MALAT1*, conferring anti-apoptotic properties in MM cells^73^.

### KDM6A

KDM6A (also known as UTX) is a JMJC demethylase which removes H3K27me2 and me3, methyl marks correlated with genomic silencing^74,75^. KDM6A regulates *HOX* genes transcription and contributes to development and differentiation, in concert with the H3K4me3 histone methyltransferase MLL2/3^74,75^. Recent studies have shown that KDM6A is implicated in cancers such as acute lymphoblastic leukemia, chronic myelomonocytic leukemia, and bladder cancer^76^.

KDM6A is also implicated in MM pathogenesis since 10% MM samples have inactivating mutations in *KDM6A*^6^. Interestingly, samples with *KDM6A* mutations do not have t(4;14) translocation, implying potential mutual exclusion of *KDM6A* mutations and *MMSET* activation^6^. Recent studies using whole-exome sequencing have also revealed *KDM6A* mutations in MM patient samples at a frequency as low as 1–3%^77,78^. Of note, *KDM6A* mutations are associated with shorter survival in MM^77^. KDM6A is supposed to be a tumor suppressor in MM, given its inactivating mutations. Indeed, loss of KDM6A has recently been shown to promote MM cell proliferation via aberrant gene repression^79^. Interestingly, significant overlap was found between genes repressed by KDM6A loss and PRC2 target genes, and EZH2 inhibitors induce cell death by reactivating these repressed genes in KDM6A-null MM cells^79^.

### KDM6B

KDM6B (also known as JMJD3) is another H3K27 demethylase which is closely related to KDM6A^75,80^. KDM6B is engaged in inflammatory response, stress-induced senescence, development, and differentiation^75,80,81^. KDM6B is also involved in the pathogenesis of cancers in a context-dependent manner^76^.

**Fig. 3:**
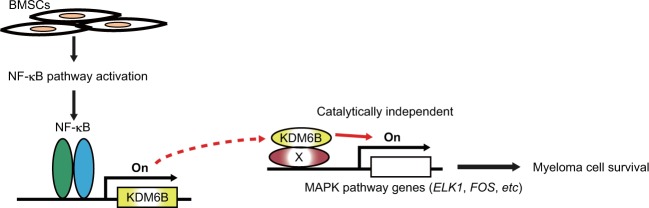
KDM6B modulates MAPK pathway independently of its demethylase activity in MM. *KDM6B* is upregulated by NF-κB, in part due to bone marrow stromal cells (BMSCs). KDM6B is in turn recruited to the loci of MAPK pathway-related genes, and activates expression of these genes in a catalytically independent manner. X represents an unknown scaffold for KDM6B

KDM6B acts as a mediator of MM cell survival in a demethylase activity-independent manner (Fig. 3)^82^. *KDM6B* expression in MM is induced by bone marrow stromal cells; conversely, this response is abrogated by IKKβ inhibitor MLN120B, indicating that *KDM6B* is activated via NF-κB signaling, a crucial survival pathway in MM cells^82^. The small hairpin RNA-mediated knockdown and CRISPR (Clustered Regularly Interspaced Short Palindromic Repeats)-mediated knockout experiments revealed that KDM6B is necessary for MM cell survival^82^. Ectopic expression of *KDM6B* partially rescues MM cells from MLN120B-induced cytotoxicity, suggesting that KDM6B is one of the downstream effectors in the NF-κB pathway in MM^82^. Of note, KDM6B is recruited to the transcriptional start sites of MAPK signaling pathway-related genes such as *ELK1* and *FOS*, and upregulates these gene expressions without altering H3K27 methylation level^82^. Overexpression of enzymatically dead *KDM6B* induces expression of MAPK pathway-related genes, indicating its catalytically independent function^82^. These data have revealed a novel function of KDM6B that links NF-κB and MAPK signaling pathways in MM.

## The roles of histone acetylation modifiers in MM

### CBP/p300

CBP and its homolog p300 form CBP/p300 family, which is recently renamed as lysine acetyltransferase 3 (KAT3) family^83^. CBP/p300 acetylates H3K18 and H3K27, activating gene expression^84^. CBP/p300 also has multiple interaction domains and serves as a molecular scaffold to recruit transcriptional complexes to transcriptional machinery^22^. These proteins also have a variety of non-histone substrates, including TP53, CREB, E2F, and MYB^22^. CBP/p300 is required for normal development, and dysregulation of its functions is implicated in hematologic malignancies including acute myeloid leukemia and T-cell acute lymphoblastic leukemia^22^.

A recent study has shown that targeting the bromodomain of CBP/p300 is a promising therapeutic strategy in MM^85^. CBP/p300 bromodomain-specific inhibitors were identified, and their biological activity was evaluated using a panel of cell lines. Interestingly, most sensitive cell lines are derived from MM. Mechanistic analyses have revealed that CBP/p300 directly activates *IRF4* expression through H3K18 and H3K27 acetylation at its super-enhancer and transcription start site; therefore, inhibition of CBP/p300 induces MM cell death via the reduction of *IRF4* expression^85^.

### HDAC1/HDAC3

**Fig. 4:**
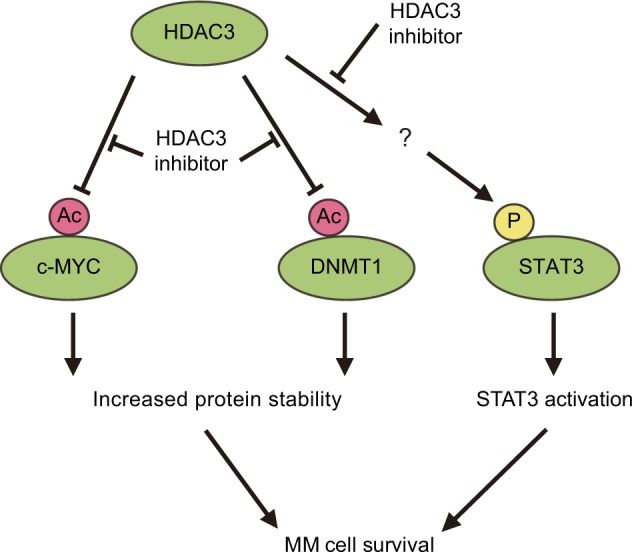
The roles of HDAC3 in MM. HDAC3 deacetylates c-MYC and DNMT1 protein, thereby stabilizing these proteins. HDAC3 also activates STAT3 via an unknown mechanism, conferring MM cell survival. HDAC3 inhibitor increases acetylation level of c-MYC and DNMT1, resulting in reduction of these proteins. HDAC3 inhibitor also decreases phosphorylated STAT3

Aberrant expression or recruitment to specific loci of HDACs has been identified in various types of cancers, and HDAC inhibitors have been shown to have anti-tumor activities, especially in hematologic cancers such as cutaneous T-cell lymphoma and myelodysplastic syndromes^86^. HDAC inhibitors also impair MM cell growth and survival. Preclinical studies have demonstrated that HDAC inhibitors trigger apoptosis, as well as induce cell cycle arrest, in MM cells^87–89^. Given that most HDAC inhibitors do not target class IIa enzymes at pharmacologically relevant concentrations^90^, class I or class IIb HDACs are implicated in MM pathogenesis. Among the class I HDACs, we have recently shown that HDAC3 is especially important in MM cell survival. Although both *HDAC1* and *HDAC3* knockdown inhibit MM cell growth, the growth inhibitory effect of *HDAC3* knockdown is more significant than *HDAC1* knockdown, in contrast to *HDAC2* knockdown with minimal effect on MM cell growth^91^. Consistent with these findings, HDAC1, 2, and 3 inhibitor MS-275 is more toxic to MM cells than HDAC1 and 2 inhibitor Merck60^91^. Furthermore, HDAC3-specific inhibitor BG45 shows a potent cytotoxic effect in MM cells^91^. Notably, knockdown or pharmacologic inhibition of HDAC3 increases acetylation level of c-MYC and DNMT1 protein^92^. Acetylation of c-MYC and DNMT1 has been shown to promote turnover of these proteins through proteasome-dependent degradation^93,94^. Indeed, increased acetylation of c-MYC and DNMT1 after *HDAC3* knockdown or inhibition enhances turnover and reduction of those proteins^92^. HDAC3 inhibition also reduces phosphorylation level of signal transducer and activator of transcription 3 (STAT3), and yet the underlying mechanism of this reduction remains elusive^91^. These data suggest that HDAC3 sustains MM cell survival via stabilization of oncoproteins c-MYC and DNMT1, as well as STAT3 activation (Fig. 4). Class I HDACs have also been shown to be the key molecules in bortezomib-induced cytotoxicity in MM^95^. Bortezomib decreases expression of class I *HDACs* via degradation of Sp1 protein, the transcriptional activator of class I *HDAC* genes^95^. Conversely, ectopic expression of *HDAC1* induces bortezomib resistance in MM cells, suggesting that class I HDACs, especially HDAC1 downregulation, mediates bortezomib-induced MM cell death^95^. Importantly, patients with higher expression of class I HDACs have significantly shorter progression-free survival^96^.

### HDAC4

Among the class IIa HDACs, HDAC4 is essential for MM cell growth and survival^97–99^. Knockdown of *HDAC4* inhibits MM cell growth via inducing apoptosis and autophagy^97–99^, whereas knockdown of *HDAC5*, *7*, and *9* does not affect MM cell growth^97^. HDAC4 acts as a transcriptional corepressor by interacting with numerous transcription factors, including MEF2 and Runx2, during development and differentiation process^100^. Interestingly, HDAC4 forms a complex with the alternative NF-κB (RelB-p52) in MM cells, and represses pro-apoptotic genes *Bim* and *BMF* via deacetylation of H3 at their promoter regions, conferring MM cell survival^99^. This study further demonstrated that disruption of the RelB-HDAC4 complex using HDAC4-mimetic polypeptide induces Bim and MM cell death^99^. HDAC4 has also been shown to downregulate a tumor suppressor miRNA miR-29b by sustaining repressive chromatin at its promoter^98^. miR-29b is known to be an ep-miRNA which targets epigenetic regulators including DNA methyltransferases^101^. Importantly, HDAC4 is also a direct target of miR-29b, forming an epigenetic negative feedback loop in MM cells^98^. Introduction of miR-29b mimics reduced HDAC4 and increased the acetylation of both histone H4 and α-tubulin, indicating that miR-29b is involved in the acetylome in MM cells^98^. HDAC4 also counteracts ER stress response in MM: knockdown of *HDAC4* under ER stress enhances ATF4 and CHOP induction, augmenting apoptosis in MM cells^97^. This result provides the rationale for combination therapy with class IIa HDAC inhibitors and proteasome inhibitors. Indeed, a class IIa HDAC inhibitor TM269 in combination with carfilzomib shows a strong synergistic cytotoxic effect in MM cell lines and patient MM cells. However, TM269 is not a clinical drug, and further studies are required for the development of clinical grade HDAC4 inhibitor^97^.

### HDAC6

Because aggregation of unfolded/misfolded proteins are toxic to cells, unfolded/misfolded proteins are tightly monitored and processed in cells. Unfolded/misfolded proteins are usually polyubiquitinated and degraded by the proteasome. However, once unfolded/misfolded proteins are overloaded and aggregated, aggresomes are formed to degrade and remove these aggregates^102^. In the context of unfolded/misfolded protein processing, a microtubule-associated deacetylase HDAC6 plays a crucial role in aggresome formation^103^. HDAC6 bridges between polyubiquitinated proteins and the dynein motor complex, thereby recruiting polyubiquitinated proteins to aggresomes^103^. Indeed, depletion of *HDAC6* results in failure of aggresome formation, and enhances induction of apoptosis in proteasome inhibitor-treated cells^103^. This is also the case in MM cells. HDAC6 inhibitor tubacin inhibits interaction of HDAC6 with dynein, and enhances bortezomib-induced cytotoxicity in MM cells^104^. Other HDAC6-specific inhibitors (ACY-1215, WT161) in combination with proteasome inhibitors also lead to synergistic accumulation of polyubiquitinated proteins and augment proteasome-induced cytotoxic effect in MM cells^105–107^. Indeed, a phase I/II trial of ACY-1215 in combination with bortezomib and dexamethasone for relapsed and refractory MM has shown promising results (described below). A recent study has suggested another HDAC6 function in MM: Imai et al.^108^ have shown that expression of *PPP3CA*, a catalytic subunit of calcineurin, is highly expressed in advanced MM patients’ samples, and that PPP3CA is indispensable for MM cell growth. HDAC6 is known to deacetylate HSP90 and maintain its chaperone function^109^. Consistent with this notion, treatment with ACY-1215 reduces the protein level of PPP3CA which is a HSP90 client protein, suggesting that HDAC6 maintains MM cell growth by preventing PPP3CA degradation^108^.

### SIRT6

SIRT6 is a chromatin-associated deacetylase which deacetylates H3K9 and H3K56, as well as non-histone protein CtIP (C-terminal binding protein (CtBP) interacting protein)^110–112^. SIRT6 confers genomic stability by promoting DNA double-strand break repair, and prevents premature cellular senescence and aging via telomere maintenance^110–112^. SIRT6 also functions as a tumor suppressor by suppressing cancer metabolism^113^.

Consistent with the notion that SIRT6 contributes to genomic stability, it confers resistance to DNA damage agents in MM^114^. SIRT6 inactivates extracellular signal-regulated kinase (ERK)/p90RSK signaling, resulting in increased DNA repairs by Chk1^114^. Indeed, either depletion of *SIRT6* or SIRT6-specific inhibitor OSS_128167 increases sensitivity to melphalan and doxorubicin in MM cells, and MEK1/2 inhibitors or RSK2 inhibitor abrogate sensitization to DNA-damaging agents in SIRT6-depleted cells^114^. A potential tumor suppressor function of SIRT6 in MM has also been shown, as knockdown of *SIRT6* stimulates MM cell growth by activating MAPK pathway^114^.

## Clinical studies of histone modifier inhibitors in MM

Among the inhibitors of histone methylation modifiers, the inhibitors of DOT1L, EZH2, and KDM1A have proceeded to phase I clinical trials for cancer therapy (https://clinicaltrials.gov). Relapsed and/or refractory MM patients were included in one of these trials, which studied the safety and clinical activity of EZH2 inhibitor GSK126 (GSK2816126), although this trial was terminated because of insufficient evidence of clinical activity (NCT02082977).

HDAC inhibitors are the most extensively investigated epigenetic drugs in clinical studies (Table 1). Among the non-selective HDAC inhibitors, romidepsin, vorinostat, and panobinostat have been well studied in MM. These HDAC inhibitors have shown a remarkable anti-MM effect in preclinical studies, and yet shown quite a modest clinical activity when used as single agents^115–117^. However, promising results have been obtained from trials in combination with other agents, especially bortezomib. In a phase I/II trial of the combination of romidepsin, bortezomib, and dexamethasone for relapsed or refractory MM, overall response rate was 60%^118^. Efficacy of vorinostat in combination with bortezomib has also been evaluated in VANTAGE trials^119,120^. In the phase III VANTAGE 088 trial, patients were randomly allocated to the vorinostat group (*n* = 315) or the placebo group (*n* = 320). Median progression-free survival was statistically longer in the vorinostat arm (7.63 months in the vorinostat group and 6.83 months in the placebo group; *p* = 0.0100), and yet this difference is not sufficient to be considered clinically relevant^120^. Different treatment schedules of vorinostat and bortezomib may further improve tolerability and efficacy of this combination. The combination of panobinostat, bortezomib plus dexamethasone has been studied in PANORAMA trials^121,122^. In the phase III PANORAMA 1 trials, patients were randomly assigned to panobinostat, bortezomib, and dexamethasone (*n* = 387), or to placebo, bortezomib, and dexamethasone (*n* = 381). Although the overall response rate did not differ between two groups (60.7% for the panobinostat group vs 54.6% for the placebo group; *p* *=* 0.09), the proportion of a complete or near complete response was significantly higher in the panobinostat group (27.6 vs 15.7%; *p* = 0.00006)^122^. Importantly, median progression-free survival was significantly longer in the panobinostat group than in the placebo group (11.99 months vs 8.08 months; *p* < 0.0001)^122^, resulting in the Food and Drug Administration (FDA) approval of panobinostat in combination with bortezomib and dexamethasone in relapsed or refractory MM. Combinations of panobinostat or vorinostat with the second-generation proteasome inhibitor carfilzomib and/or immunomodulatory drugs (IMiDs) have also been investigated in phase I/II studies, and have shown promising results^123–125^.

**Table 1:** HDAC inhibitors studied in clinical trials in MM

Name	Chemical structure	Specificity	Clinical trial in MM (phase)
Romidepsin (FK228)	Cyclic peptide	Class I HDACs	I/II
Vorinostat (SAHA)	Hydroxamic acids	Class I, II, IV HDACs	III
Panobinostat (LBH589)	Hydroxamic acids	Class I, II, IV HDACs	III (FDA approved)
Quisinostat (JNJ26481585)	Hydroxamic acids	Class I, II, IV HDACs	I
Givinostat (ITF2357)	Hydroxamic acids	Class I, II HDACs	II
CKD-581	Hydroxamic acids	Class I HDACs	I
Belinostat (PXD101)	Hydroxamic acids	Class I, II, IV HDACs	II
Abexinostat (PCI-24781)	Hydroxamic acids	Class I, II HDACs	I
Fimepinostat (CUDC-907)	Hydroxamic acids	Class I, II HDACs+PI3K	I
Tinostamustine (EDO-S101)	Hydroxamic acids	Bendamustine–vorinostat fusion	I
Ricolinostat (ACY-1215)	Hydroxamic acids	HDAC6	I/II
Citarinostat (ACY-241)	Hydroxamic acids	HDAC6	I
Entinostat (MS-275)	Benzamide	Class I HDACs	I
Tacedinaline (CI-994)	Benzamide	Class I HDACs	II
AR-42	Benzamide	Class I, II, IV HDACs	I
4SC-202	Benzamide	Class I HDACs	I
CXD101	Benzamide	Class I HDACs	I

In the context of the use of non-selective HDAC inhibitors, a relatively high frequency of side effects, including diarrhea and thrombocytopenia, are observed, which limits dose and time of treatment, especially in combination therapy with other agents. Based on this background, selective HDAC inhibitors are under development to reduce adverse effects, keeping anti-tumor activity. Indeed, the first selective HDAC6 inhibitor ACY-1215 (ricolinostat) has been examined as a single agent or in combination with bortezomib and dexamethasone for relapsed or refractory MM in a phase I/II study^126^. Although single-agent ACY-1215 therapy resulted in no clinical responses similar to non-selective HDAC inhibitors, 37% of the overall response was observed in combination therapy with daily ACY-1215 at ≥160 mg^126^. Combination therapy at an ACY-1215 dose of 160 mg daily was well tolerated, with less severe adverse effects compared with published data on non-selective HDAC inhibitors, suggesting that selective inhibition of HDAC6 is promising in MM treatment^126^. ACY-1215 has also been examined in combination with lenalidomide and dexamethasone in relapsed or refractory MM^127^. In this multicenter phase Ib trial, ACY-1215 with lenalidomide and dexamethasone has been shown to be safe and well tolerated, with an overall response rate of 55%^127^. Further clinical studies with HDAC6 inhibitor are ongoing (NCT02189343, NCT01997840, NCT02400242).

## Conclusions and perspectives

Accumulating studies have revealed the biological importance of histone modifiers in MM. Histone modifiers contribute to the pathogenesis of MM by mediating modifications not only of histone but also of non-histone proteins; as well as by catalytically independent functions. In this context, epigenetic drugs targeting histone modifiers are now being developed. Of note, an HDAC inhibitor panobinostat is already available for patients with refractory/relapsed MM. However, further studies are required to more comprehensively understand the roles of histone modifiers and develop related novel therapeutics in MM. The genome-wide locations of each of histone modifiers and histone modifications have to be defined. The methylome and acetylome analyses will identify novel non-histone substrates for histone modifiers. These basic studies will enable us to understand the more precise mechanisms whereby targeted therapies induce anti-MM activities, and also provide the rationale for combination therapies. While there is a need to develop selective inhibitors of each enzyme to reduce unfavorable adverse effects, this may be difficult due to structural homology within the catalytic domains between family proteins. Nevertheless, small-molecule inhibitors specific for EZH2 and HDAC6 have already been developed and proceeded to clinical trials, highlighting the potential of epigenetic therapies to improve patient outcome in MM.
