# Author Correction: The biological significance of histone modifiers in multiple myeloma: clinical applications

**DOI:** 10.1038/s41408-021-00559-4

**Published:** 2021-10-06

**Authors:** Hiroto Ohguchi, Teru Hideshima, Kenneth C. Anderson

**Affiliations:** 1 Jerome Lipper Multiple Myeloma Center, Department of Medical Oncology, Dana-Farber Cancer Institute, Harvard Medical School, Boston, MA USA; 2 Division of Disease Epigenetics, Institute of Resource Development and Analysis, Kumamoto University, Kumamoto, Japan

**Keywords:** Translational research, Myeloma

Correction to: *Blood Cancer Journal* 10.1038/s41408-018-0119-y, published online 22 August 2018

It came to the author’s attention that the conflict of interest statement is incomplete, the revised COI statement is as follows:

**Conflict of interest**

H.O. and T.H. declare that they have no conflict of interest. K.C.A. serves on advisory boards for Celgene, Millennium, and Gilead Sciences, and is a scientific founder with financial interest in Acetylon, OncoPep, and C4 Therapeutics.

